# Unveiling the Genetic Basis Underlying Rice Anther Culturability via Segregation Distortion Analysis in Doubled Haploid Population

**DOI:** 10.3390/genes14112086

**Published:** 2023-11-17

**Authors:** Bin Sun, Xiaorui Ding, Junhua Ye, Yuting Dai, Can Cheng, Jihua Zhou, Fuan Niu, Rongjian Tu, Qiyan Hu, Kaizhen Xie, Yue Qiu, Hongyu Li, Zhizun Feng, Chenbing Shao, Liming Cao, Anpeng Zhang, Huangwei Chu

**Affiliations:** 1 Key Laboratory of Germplasm Innovation and Genetic Improvement of Grain and Oil Crops (Co-Construction by Ministry and Province), Ministry of Agriculture and Rural Affairs, Crop Breeding and Cultivation Research Institute, Shanghai Academy of Agricultural Sciences, Shanghai 201403, China; sxb0708@126.com (B.S.); yejunhua@saas.sh.cn (J.Y.); daiyuting@saas.sh.cn (Y.D.); chengcan@saas.sh.cn (C.C.); zhoujihua@saas.sh.cn (J.Z.); niufuan@saas.sh.cn (F.N.); turongjian@hotmail.com (R.T.); clm079@163.com (L.C.); 2 Shanghai Key Laboratory of Agricultural Genetics and Breeding, Shanghai Academy of Agricultural Sciences, Shanghai 201106, China; 3 Development Center of Plant Germplasm Resources, College of Life Sciences, Shanghai Normal University, Shanghai 200234, China; dxr20000209@163.com (X.D.); qiuyue0124@outlook.com (Y.Q.); 4 College of Fisheries and Life Science, Shanghai Ocean University, Shanghai 201306, China; huqiyan1999@163.com (Q.H.); 19862189660@163.com (H.L.); 5 MOE Key Laboratory of Crop Physiology, Ecology and Genetic Breeding, College of Agronomy, Jiangxi Agricultural University, Nanchang 330045, China; 18621311381@163.com (K.X.); bulinglingoo@163.com (C.S.); 6 College of Agronomy, Shanxi Agricultural University, Jinzhong 030801, China; f164138146@163.com

**Keywords:** anther culture, doubled haploid, rice, segregation distortion

## Abstract

Anther culture (AC) is a valuable technique in rice breeding. However, the genetic mechanisms underlying anther culturability remain elusive, which has hindered its widespread adoption in rice breeding programs. During AC, microspores carrying favorable alleles for AC are selectively regenerated, leading to segregation distortion (SD) of chromosomal regions linked to these alleles in the doubled haploid (DH) population. Using the AC method, a DH population was generated from the *japonica* hybrid rice Shenyou 26. A genetic map consisting of 470 SNPs was constructed using this DH population, and SD analysis was performed at both the single- and two-locus levels to dissect the genetic basis underlying anther culturability. Five segregation distortion loci (SDLs) potentially linked to anther culturability were identified. Among these, *SDL5* exhibited an overrepresentation of alleles from the female parent, while *SDL1.1*, *SDL1.2*, *SDL2*, and *SDL7* displayed an overrepresentation of alleles from the male parent. Furthermore, six pairs of epistatic interactions (EPIs) that influenced two-locus SDs in the DH population were discovered. A cluster of genetic loci, associated with EPI-1, EPI-3, EPI-4, and EPI-5, overlapped with *SDL1.1*, indicating that the *SDL1.1* locus may play a role in regulating anther culturability via both additive and epistatic mechanisms. These findings provide valuable insights into the genetic control of anther culturability in rice and lay the foundation for future research focused on identifying the causal genes associated with anther culturability.

## 1. Introduction

Anther culture (AC) is an extremely effective technique employed in rice breeding [1,2]. In traditional rice breeding, diverse varieties with desired traits are crossed and subsequently subjected to 6–9 generations of self-pollination to obtain near-homozygous inbred lines. This time-consuming process enables the generation of inbred lines that pyramid desired traits from different parental lines [3]. AC enables the production of homozygous doubled haploid (DH) plants from the haploid microspores of heterozygous plants via in vitro culture in a single generation [4,5]. As a result, it significantly reduces the time and costs associated with developing breeding lines, making it highly promising for enhancing overall breeding efficiency.

AC encompasses several essential steps, which include callus induction, plant regeneration, and chromosome doubling [6]. The effectiveness of AC can be influenced by multiple factors, such as the genotype and growth environment of the plant, the developmental stage of the anthers, the cold pretreatment of the explant, the nutrient composition and hormone levels of the culture medium, as well as the specific conditions employed during the anther culture process [2,4,6,7,8,9,10]. Among these factors, the genotype is the main determinant of anther culturability. Previous studies have consistently shown that cultivated rice displays a greater capacity for anther culture when compared to wild rice [11]. Furthermore, within cultivated rice varieties, *japonica* rice has been found to exhibit significantly higher efficiency in anther culture compared to *indica* rice [12,13,14,15].

However, the genetic basis underlying anther culturability in rice remains largely unknown, posing as one of the major obstacles hindering the widespread adoption of AC techniques in rice breeding. Anther culturability in rice is a complex quantitative trait controlled by multiple nuclear genes [16,17]. Thus far, only a limited number of quantitative trait loci (QTLs) linked to anther culturability have been identified, primarily due to the challenges involved in obtaining reliable phenotypic data. He et. al. analyzed the QTLs responsible for four key traits associated with anther culturability: callus induction frequency (CI), green plantlet differentiation frequency (GPD), albino plantlet differentiation frequency (APD), and green plantlet yield frequency (GPY). They identified five QTLs for CI, two for GPD, and a significant QTL for APD [18]. Another recent study by Huang et. al. identified a total of eight QTLs for anther culturability across three different environments [11]. However, to the best of our knowledge, the specific gene responsible for anther culturability has not been characterized yet. Therefore, the urgent and unresolved challenge remains in the identification of further genetic loci and the characterization the specific genes associated with anther culturability in rice.

It is hypothesized that, in the process of anther culture, microspores carrying favorable alleles for improved anther culturability are selectively preferred to generate DH plants [19]. Consequently, DH populations represent a distinct population that undergoes gametic selection, resulting in shifts in the frequencies of genes governing anther culturability [20,21]. Molecular markers linked to these genes exhibit concordant frequency shifts [22,23]. Therefore, the mapping of segregation distortion loci (SDLs) stands as a robust alternative to mapping QTLs associated with anther culturability, offering increased efficacy in the identification and characterization of these genetic loci.

The present study aimed to identify the additive or epistatic genetic loci that are potentially responsible for rice anther culturability via segregation distortion analysis at both single- and two-locus level using a DH population.

## 2. Materials and Methods

### 2.1. Materials

In this study, the experimental materials consisted of a DH population comprising 234 lines. The DH population was generated through anther culture of Shenyou 26, a three-line hybrid japonica rice cultivar developed by the Shanghai Academy of Agricultural Sciences in 2016. Shen 9A was utilized as male-sterile line. Shen 9B, the maintainer line, was used to pollinate Shen 9A for the purpose of propagating Shen 9A. Shenhui 26, the restorer line, was employed to pollinate Shen 9A, resulting in the production of hybrid Shenyou 26. The F_2_ population was generated through the self-pollination of the hybrid Shenyou 26.

### 2.2. Anther Culture

Shenyou 26 panicles at the late microspore uninucleate stage were collected for anther culture. At the late microspore uninucleate stage, young panicles are still enclosed in leaf sheaths. During the sampling process, the panicles, along with their leaf sheaths, were carefully plucked from the plants. To ensure cleanliness, the surface of the leaf sheaths was disinfected by wiping them with an alcohol-soaked cotton ball. Subsequently, the panicles were wrapped in moist gauze and then covered with cling film to prevent dehydration. These wrapped samples underwent a 12-day cold treatment at 8 °C.

After removing the leaf sheaths, the panicles were surface sterilized with 15% sodium hypochlorite and rinsed eight times with sterile water. The sterilized florets were dissected to retrieve the anthers, which were subsequently cultured on Chu N6 medium [24] supplemented with 2 mg/L 2,4-D, 1 mg/L NAA, 1 mg/L KT, 0.5 g/L casein hydrolysate, 30 g/L sucrose, and 3.5 g/L phytagel. Cultures were placed in darkness at 26 °C to induce callus formation.

After about 20 days of culture, calluses formed on the anthers. Once the calluses reached 2–3 mm in size, they were transferred to Murashige and Skoog (MS) medium [25] supplemented with 2 mg/L 6-BA, 0.5 mg/L NAA, 2.5 mg/L MET, 0.5 g/L casein hydrolysate, 30 g/L sucrose, and 3.5 g/L phytagel and cultured at 26 °C with a 14 h light photoperiod (100 µmol m^−2^ s^−1^) and 10 h of darkness. After approximately one week of cultivation, the calluses began to generate green buds which eventually developed into young plantlets. Subsequently, the regenerated green plantlets were moved to 1/2 MS medium with 10 g/L sucrose and 3.5 g/L phytagel to promote root development.

### 2.3. Preparation of Genotyping-by-Sequencing (GBS) Libraries

The GBS libraries were prepared following the protocol developed by Qi et al. [26]. In brief, genomic DNA (200 ng) from each DH line and its parents was digested with MspI and PstI-HF (New England Biolabs, Ipswich, MA, USA). An additional 20 min incubation at 75 °C was performed to inactivate the restriction enzymes. The 20 µL restriction digest was then mixed with 1 µL of barcoded PstI-HF adapter (0.1 µM), 1.5 µL of common MspI adapter (0.1 µM), 4 µL of 10 × T4-DNA ligase buffer, and 200 U of T4 DNA ligase (New England Biolabs, Ipswich, MA, USA) in a total volume of 40 µL. Ligation was conducted at 22 °C for 2 h.

Following ligation, fragments smaller than 300 bp were removed by incubating the samples with 0.7 volumes of Sera-Mag SpeedBeads (GE Healthcare Life Sciences, Uppsala, Sweden) at room temperature for 5 min. The beads were then separated from the supernatant using a magnetic stand and washed three times with 200 µL of freshly prepared 70% ethanol. DNA was eluted from the air-dried beads with 40 µL of 10 mM Tris-HCl (pH 8.0). From the resulting eluate, 3 µL was added to a cocktail containing 16 µL of H_2_O, 5 µL of 5× Taq master mix (New England Biolabs, Ipswich, MA, USA), 0.5 µL of forward primer specific to the barcoded adapter (10 µM), and 0.5 µL of reverse primer with homology to the common adapter (10 µM). PCR amplification was performed for each sample separately, with an initial denaturation at 95 °C for 30 s, followed by 16 cycles of denaturation at 95 °C for 30 s, primer annealing at 62 °C for 20 s, and fragment elongation at 68 °C for 15 s. A final fragment elongation step at 68 °C for 5 min was included.

To check the PCR product, 8 µL was loaded onto a 1.5% agarose gel. The DNA concentration of each GBS library was measured using a Qubit 2.0 instrument and the Qubit™ dsDNA HS assay kit. Only GBS libraries with concentrations >5.0 ng/µL were selected for sequencing. For pooling, 30 ng of each GBS library was combined and subjected to the removal of primers, dNTPs, and small DNA fragments using 0.7 volumes of Sera-Mag SpeedBeads. The pooled GBS libraries (100 ng) were sequenced on an Illumina Nova platform with paired-end 150 bp reads.

Illumina raw sequence reads were demultiplexed and split by barcode using the ‘process_radtags’ module within the ‘Stacks’ program (v2.4) [27]. Forward reads were retained if they contained both the barcode and the PstI restriction site. The quality of the reads was assessed using fastp (v0.20.0) [28]. The clean reads were aligned to MSU Rice Genome Annotation Project Release 7 [29] using BWA-MEM (v0.7.17) with default parameters [30].

### 2.4. SNP Calling and Genetic Map Construction

SNP calling was performed using the Genome Analysis Toolkit (GATK) [31]. Samples with a SNP homozygous ratio below 80% were excluded from further analysis. Heterozygous genotypes were considered missing data. To ensure reliable results in subsequent analyses, SNP filtering was conducted using VCFtools (v0.1.16) [32] with the following criteria: (1) Loci with a sequencing depth of less than 4 were excluded. (2) SNPs with a minor allele frequency (MAF) of less than 0.01 were filtered out. (3) SNPs missing in more than 20% of the samples were removed.

The order of SNP markers along the chromosome was determined based on the physical positions of SNPs in the MSU Rice Genome Annotation Project Release 7 [29]. The Kosambi mapping function was employed to convert the recombination frequency into genetic distances of the SNP markers. Redundant markers were removed using the BIN functionality. Each bin retained the marker with the lowest number of missing data points. Finally, the retained markers were utilized to construct the genetic linkage map using the MAP functionality in QTL IciMapping v4.1 [33].

### 2.5. Analysis of Segregation Distortion Loci (SDLs)

The SDL analysis was conducted using the interval mapping of additive and dominant (IM-ADD) method with the SDL functionality in QTL IciMapping v4.1 [33]. A step size of 1.0 cM was adopted for the IM-ADD method. The significance of the presence of SDLs was determined by setting the logarithm of odds (LOD) threshold value at 3.5.

### 2.6. Segregation Ratio Analysis of SDLs and Rf-1 Loci in F_2_ Populations

Based on a comparative genomic sequence between Shen 9A and Shenhui 26, Indel markers were designed in proximity to SDLs. Additionally, a codominant Indel marker was designed, targeting a 574 bp deletion in the non-allele *Rf1a* gene within the *Rf-1* locus [34,35]. An F_2_ population comprising 96 individuals was established, and genomic DNA was extracted from leaf tissues using the CTAB method. PCR amplification was performed to determine the genotypes of each individual at the SDL loci and the *Rf-1* locus. The primer sequences and amplified fragment lengths for the Indel markers utilized can be found in Table 1.

### 2.7. Identification of Epistatic Interactions between Two Loci Causing SD

The methodology used to identify two-locus epistatic interactions followed the procedures outlined in previous studies [36,37]. In summary, a Chi-square independence test was conducted using the chisq.test function in R studio to evaluate all combinations of SNP pairs across the entire genome and explore potential combinations of epistatic interaction loci. To account for the large number of tests conducted and avoid type I errors, *p*-values were adjusted using the p.adjust function in R studio with the Benjamini–Hochberg procedure for false discovery rate (FDR) correction. SNP pairs that showed significant associations (*p* < 0.001) and had more than three consecutive linked SNPs at each locus were considered indicative of two-locus epistatic interactions associated with SD.

## 3. Results

### 3.1. GBS Sequencing of the DH Population

**Figure 1:**
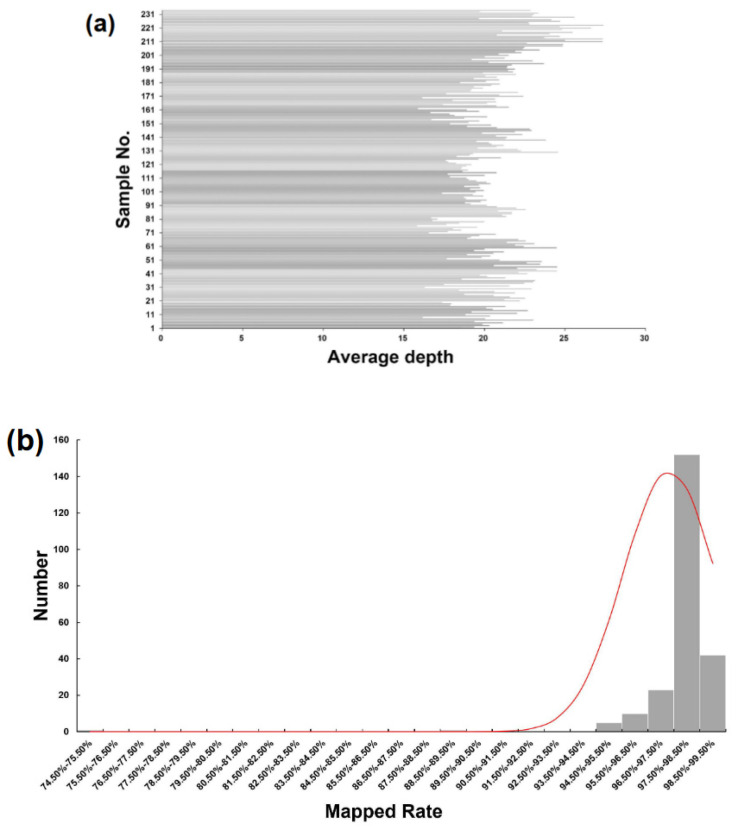
GBS sequencing of the DH population. (**a**) Sequencing depths of 232 samples. The x-axes indicate sequencing depth and the y-axes indicate individual samples. (**b**) Frequency distribution of the percentage of raw reads of each sample mapped to the reference genome.

The GBS library was constructed using a total of 232 DH lines and 2 parental lines, Shenyou 26 and Shen 9A. The library was sequenced using the Illumina Nova platform, resulting in a total of 1,009,240,248 raw reads, with an average sequencing depth of 20.64. Figure 1a shows the sequencing depth details of the 234 samples. After removing low-quality reads, 951,043,496 clean reads were aligned to the MSU Rice Genome Annotation Project Release 7 reference genome sequence. Out of these, 929,603,456 (97.75%) clean reads aligned to unique positions. The average number of mapped reads per sample was 3,972,664, with a median of 3,899,039 (Figure 1b and Supplementary Table S1).

### 3.2. SNP Calling and Linkage Map Construction

**Figure 2:**
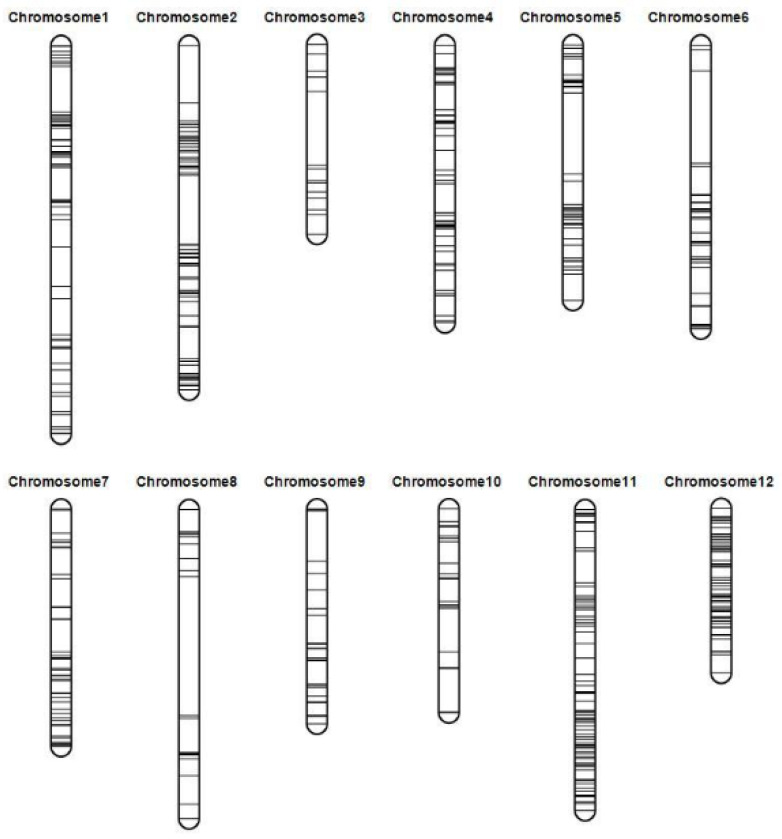
Linkage map constructed using the genotyping-by-sequencing approach. The markers are indicated by horizontal lines. The genetic distance in centimorgans (cM) between flanking markers was determined using the Kosambi mapping function.

After performing SNP calling, eight samples were identified as failed samples due to SNP homozygous ratios below 80% (Supplementary Table S2). Consequently, these samples were excluded from further analysis. The final analysis cohort consisted of 226 samples, which included the 2 parental lines and 224 DH lines. After SNP filtering, a total of 6659 high-quality SNP markers (Supplementary Table S3) were obtained. Adjacent markers with the same genotype across the entire DH population were considered a single bin. The markers with the fewest missing data points in each bin were retained. Ultimately, 470 markers were retained for the construction of the linkage map (Supplementary Table S4). The final linkage map had a total length of 1650.94 cM. The genetic length of individual chromosomes ranged from 88.10 to 198.93 cM, with an average distance between adjacent markers of 4.23 cM. A total of five intervals had genetic distances larger than 30 cM (Figure 2 and Table 2).

### 3.3. Mapping of Segregation Distortion Loci (SDLs)

**Figure 3:**
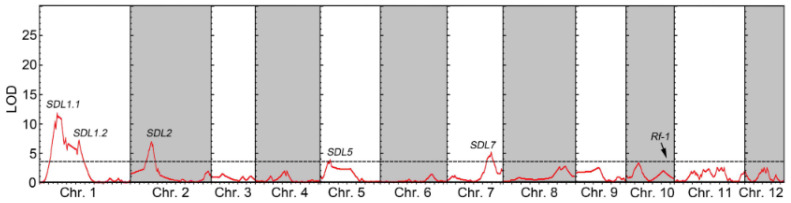
SDLs detected in the DH population are shown. The x-axis represents the physical position along each chromosome, while the y-axis represents the logarithm of odds (LOD). A horizontal dotted line indicates the LOD declaration threshold (3.5).

SDL analysis was conducted to identify the genetic loci that could be linked to anther culturability. A total of five significant SDLs were identified, namely *SDL1.1*, *SDL1.2*, *SDL2*, *SDL5*, and *SDL7*. Among them, two SDLs were found on chromosome 1, while one SDL each was located on chromosomes 2, 5, and 7 (Figure 3 and Table 3). Except for a significant bias towards the allele from female parent Shen9A at the *SDL5* locus, the other four loci were overrepresented in alleles from male parent Shenhui 26 (Supplementary Table S5). This indicates that microspores with Shen9B alleles at the SDL5 locus are more likely to produce doubled haploid (DH) offspring through anther culture compared to microspores with Shenhui 26 alleles. On the other hand, for the *SDL1.1*, *SDL1.2*, *SDL2*, and *SDL7* loci, microspores with Shenhui 26 alleles are more likely to generate DH offspring through anther culture.

### 3.4. Segregation Ratio of SDLs in F_2_ Population

SD is commonly observed in F_2_ progenies resulting from crosses of various plants, and it is widely acknowledged as a significant contributing factor to the formation of reproductive barriers [36,37]. Several biological processes, such as the non-random segregation of gametes during meiosis, post-meiotic gamete dysfunction or differential gamete success, and differential zygotic fitness, may give rise to SD in F_2_ populations [38]. DH lines obtained through rice anther culture are derived from post-meiotic microspores at the mid-uninucleate stages. Therefore, we believe that factors such as non-random segregation of gametes during meiosis could also lead to SD in DH populations.

To investigate whether the SDLs identified in this study were caused by meiotic gametophyte selection during anther development, we analyzed the genotype frequency distribution of each SDL in the F_2_ population derived from the cross between Shen 9A and Shenhui 26. The analysis results indicated that the segregation ratios of the *SDL1.1*, *SDL1.2*, *SDL2*, *SDL5*, and *SDL7* loci in the F_2_ population followed the 1:2:1 Mendelian ratio of diploid genotypes (Table 4), suggesting that the segregation distortion of these SDLs in the DH population may be caused by the in vitro anther culture process and is likely associated with anther culturability.

In this study, the female parent, Shen 9A, was a Boro II (BT)-type cytoplasmic male sterility (CMS) line. The BT-type cytoplasm contains a mitochondrial gene called *orf79*, which encodes a cytotoxic peptide resulting in male sterility. Meanwhile, the male parent, Shenhui 26, was an elite restorer line with a functional *Rf-1* locus, responsible for restoring male fertility by suppressing the production of the ORF79 peptide [34,39]. Analysis of the segregation of the *Rf-1* locus in the F2 population indicated a deviation from the expected 1:2:1 Mendelian ratio to a 1:1 ratio (Table 4). Interestingly, no significant segregation bias was observed at the *Rf-1* locus in the DH population derived from the same hybrids (Figure 3). This finding suggests that microspore with BT cytoplasm carrying the non-functional *Rf-1* allele could successful regenerate into seedlings through in vitro anther culture, although it would undergo abortion during in vivo pollen maturation.

### 3.5. Two-Locus Epistatic Interaction (EPI) Causing SD in DH Population

**Figure 4:**
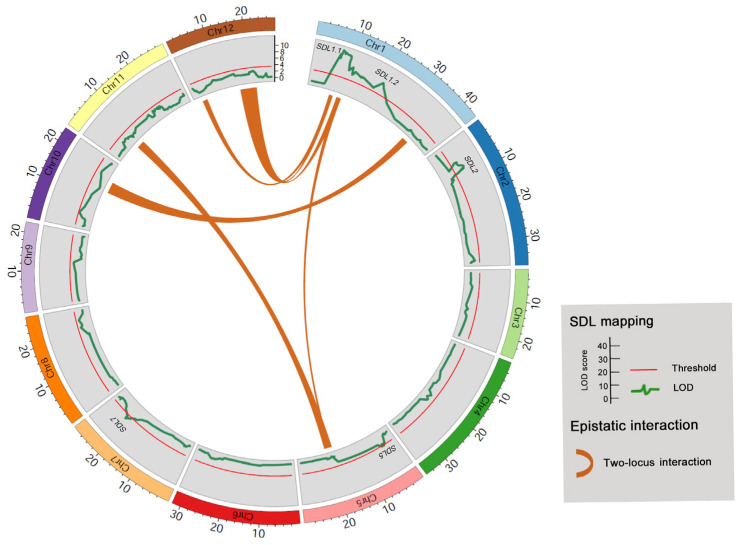
Illustration of the comparison between two-locus SDLs and single-locus SDLs in the DH population. Chi-square test was conducted to identify two-locus epistatic interactions leading to SD.

According to Mendel’s Law, in biparental-derived DH populations, if two genetic loci are independent, there are four possible genotypic combinations. The expected segregation ratio for these four genotypes is 1:1:1:1. However, when these two loci exhibit epistatic interactions (EPIs) that affect rice anther culture, it can lead to two-locus SDs in the DH population. To assess the presence of EPIs affecting rice anther culture, we investigated the two-locus SD in the DH population. As a result, a total of four pairs of EPIs were identified, involving all chromosomes except for chromosomes 3, 4, 7, and 10 (Figure 4 and Table 5). This observation suggests that EPIs may have a substantial impact on SD in the rice DH population at the whole-genome level, thus highlighting the complex genetic control mechanism underlying rice anther culturability. It is worth noting that a cluster of genetic loci, associated with *EPI-1*, *EPI-3*, *EPI-4*, and *EPI-5*, appeared to overlap with *SDL1.1*, which displayed strong SD at the single-locus level (Figure 4 and Table 3). This finding suggests that the *SDL1.1* locus may play a role in regulating anther culturability through both additive and epistatic mechanisms.

To delve deeper into the impact of EPIs on anther culturability, we performed a comprehensive analysis of the segregation frequencies of genotypic combinations involving each EPI locus (Table 6). For the two loci involved in epistatic interaction EPI-1, the genotype combination I/II exhibited the highest frequency. Similarly, for EPI-2, the genotype combination i/II displayed the highest frequency. In the cases of EPI-3, EPI-4, and EPI-5, the genotype combination I/III showed the highest frequencies. Conversely, for EPI-6, the genotype combination i/ii exhibited the highest frequency (Table 6). These findings suggest that microspores carrying these epistatic genotype combinations may have an advantageous effect on anther culturability.

## 4. Discussion

In selected populations, the frequencies of genes controlling the target traits may deviate from Mendelian segregation ratios [22]. These deviations could potentially contain vital genetic information relevant to the selected target traits. The effectiveness of mapping QTLs through the identification of segregation distortion (SD) in selected populations has been demonstrated [23]. In a recent study, the SD approach was utilized to detect the genetic factors influencing haploid male fertility (HMF) in maize, and the researchers successfully identified four QTLs, *qhmf1*, *qhmf2*, *qhmf3*, and *qhmf4*, in the selected haploid population, which was generated through in vivo haploid induction [40], further emphasizing the reliability and utility of SD detection as an approach for QTL mapping in selected populations.

It is speculated that during the anther culture process in rice, microspores that are carrying favorable alleles for anther culture are selectively preferred for response to anther culture, resulting in an overrepresentation of beneficial alleles in the DH populations [41]. Therefore, the genetic basis underlying anther culturability can be unveiled by analyzing the SDL loci present in DH populations. SD is frequently observed in anther culture-derived doubled haploid (DH) populations of rice, as evidenced by analyzing the segregation ratios of genetic markers in the DH populations [19,20,21,41,42,43,44]. These studies have revealed that SD occurs across almost all chromosomes, highlighting the intricate genetic regulatory mechanism underlying anther culturability in rice.

In this study, we identified a total of five SDLs, with only *SDL5* showing a preference for the allele of the female parent, Shen9A, while the remaining four loci exhibited a bias towards the allele of the male parent, ShenHui26. As DH lines are generated from post-meiotic microspores through in vitro culture, it is reasonable to hypothesize that factors such as non-random segregation of gametes during meiosis, which will result in SD in the F_2_ generation, may also cause the occurrence of SD in DH populations. To explore whether the SDL loci identified in this study were influenced by gamete selection during meiosis in the anther, we conducted an analysis of the segregation ratios for these five SDL loci in the F2 populations derived from the same hybrid. The results showed that all five loci exhibited a Mendelian segregation pattern of 1:2:1 in the F2 populations (Table 4). This suggests that these loci are likely affected by gamete selection during the in vitro anther culture process and are associated with anther culturability.

*Rf-1* is the genetic locus in the restorer line that is essential for restoring the BT-type CMS and is widely utilized in the production of hybrid rice varieties for commercial purposes [35]. In hybrids, around 50% of microspores lack the functional *Rf-1* locus, leading to pollen sterility [39,45], which could cause the segregation of the *Rf-1* locus to deviate from the expected 1:2:1 Mendelian ratio in F2 populations (Table 4), implying an atypical inheritance pattern of *Rf-1* loci in the progeny of hybrids. Unexpectedly, in the DH population derived from the same hybrid rice, the segregation ratio of the *Rf-1* locus conformed to a Mendelian ratio of 1:1 (Figure 3). This result indicates that in hybrid rice, even in the absence of *Rf-1*, the presence of the mitochondrial gene *orf79*, which encodes cytotoxic peptides, in the microspores does not affect their competency to anther culture. This may be attributed to the fact that pollen abortion occurs at the trinucleate stage in the CMS-BT line [46], whereas microspores exhibit the highest competency for anther culture at the uninucleate stage and lose their competency at the binucleate or trinucleate stages [47].

Anther culturability is a complex trait that is controlled by multiple genetic loci, which has been demonstrated through analysis of diallel crosses [16,17]. The identification of QTLs associated with anther culturability further strengthens this conclusion [11,18]. It is worth noting that, apart from additive QTLs, epistatic effects, representing non-linear interactions between alleles at different loci, can also have an impact on quantitative traits [48]. The epistatic interaction (EPI) analysis conducted in this study revealed the existence of six pairs of EPIs, which contributed to two-locus segregation distortions in the DH population. This finding suggests that anther culturability is a complex quantitative trait influenced not only by additive effects at specific loci but also by the effects of epistatic interactions.

The identification of QTLs and the characterization of functional genes associated with anther culturability pose significant challenges, primarily due to the difficulty in accurately quantifying the traits of anther culturability [11,18]. In this study, we utilized an SD analysis approach and successfully identified five SDLs and six pairs of EPIs that may potentially contribute to anther culturability. The subsequent construction of introgression lines through backcrossing and fine-mapping conducted on these SDLs and EPIs may provide a promising avenue for the characterization of functional genes involved in regulating anther culturability.

Asian cultivated rice is composed of two distinct subspecies, namely indica and *japonica*, which exhibit differences in various developmental and physiological characteristics [49]. While the anther culture technique has proven effective for breeding *japonica* rice, its applicability to *indica* rice is still constrained, primarily by its inherently recalcitrant genetic background [50]. The genetic loci identified in this study, which are associated with anther culturability, provide a possibility for introducing favorable alleles of these loci into the core varieties of *indica* rice. This will facilitate the application of anther culture methods in *indica* rice breeding and is expected to greatly enhance the efficiency and quality of the breeding process for *indica* rice.

In summary, the findings of this study provide a solid foundation for future investigations aimed at unraveling the key genes that regulate anther culturability. Moreover, the molecular markers linked to SDLs hold great potential in identifying varieties with high anther culturability for DH line production and the breeding of elite hybrids.

## 5. Conclusions

In this study, we employed the segregation distortion approach to identify 5 SDLs in a DH population composed of 234 lines derived from the 3-line japonica hybrid rice variety Shenyou 26. Notably, only *SDL5* exhibited a deviation towards the female parent, Shen 9A, while the remaining four SDLs, namely *SDL1.1*, *SDL1.2*, *SDL2*, and *SDL7*, displayed a bias towards the male parent, Shenhui 26. Furthermore, six pairs of EPIs contributing to two-locus segregation distortion were identified. Interestingly, the *SDL1.1* locus exerted an influence on SD at both the single-locus and two-locus levels. These findings provide valuable insights into the genetic control mechanisms underlying anther culturability.

## Figures and Tables

**Table 1:** The primers of Indel markers used in this study.

Locus	Marker Name	Forward Primer (5′-3′)	Reversed Primer (5′-3′)	Fragment Length (bp)
*SDL1.1*	Chr1-7062962	TGAGGGAGCAAAAGTCGTGTA	AGTCTTGCTTGAGCCTTTTCT	109 (Shen9A)/96 (Shenhui26)
*SDL1.2*	Chr1-20652344	GCCACCACGTATAGTACCACC	ATGCTACACCGTACTGTTTATTGG	173 (Shen9A)/161 (Shenhui26)
*SDL2.1*	Chr2-6769846	ACGAACCCCAAGACATCACTC	TGAGATCTGTATTTTAAGGACTCCA	108 (Shen9A)/85 (Shenhui26)
*SDL2.2*	Chr2-33964543	TATGCGTTAGTTCGTGCGTC	AGATGTGATCAAACTTGTCTAAGGA	100 (Shen9A)/84 (Shenhui26)
*SDL5*	Chr5-3034981	GTTCGGGAACGTGGTTCAAA	AGCACGGCCATCCTCATTTC	83 (Shen9A)/91 (Shenhui26)
*SDL7*	Chr7-21343661	CGGATTGAGAGTGGCGTTTG	ATGGCCCCTTGGAACTGAAG	108 (Shen9A)/97 (Shenhui26)
*Rf-1*	*Rf1aM*	GGACCGGGGGATTTTACCTG	AACCCAACTGAGACCATGCC	383 (Shen9A)/957 (Shenhui26)

**Table 2:** Distribution of genetic markers across the 12 chromosomes in rice.

Chromosome	No. of Markers	Genetic Length (cM)	Average Interval (cM)	Max. Interval Length (cM)	Average Physical Distance (kb)
1	56	198.93	3.55	23.39	765.05
2	57	177.28	3.11	35.50	626.61
3	14	100.91	7.21	39.19	1494.75
4	46	144.13	3.13	15.04	705.83
5	37	133.40	3.61	42.42	796.40
6	31	147.47	4.76	47.90	968.72
7	40	124.43	3.11	17.08	690.00
8	19	159.97	8.42	71.93	1408.65
9	24	113.08	4.71	26.59	876.16
10	20	107.73	5.39	23.22	1113.89
11	73	155.51	2.13	16.50	391.05
12	53	88.10	1.66	9.95	485.08
Overall	470	1650.94	4.23	30.72	860.18

**Table 3:** *SDL*s identified in the DH population and Chi-square test of flank markers.

Locus	Position (cM)	LOD Value	Range (cM)	Left Marker		Right Marker	
				Name	χ^2^ Test (−log10*P*)	Name	χ^2^ Test (−log10*P*)
*SDL 1.1*	39	11.66	39.0–40.3	Chr1-7030821	12.17	Chr1-7624356	11.75
*SDL 1.2*	87	7.07	86.6–89.3	Chr1-20457965	7.97	Chr1-21236975	6.67
*SDL 2.1*	46	6.87	44.3–46.5	Chr2-6765899	7.31	Chr2-7165431	7.64
*SDL 5*	18	3.74	15.7–18.0	Chr5-2465095	5.00	Chr5-3055104	4.32
*SDL 7*	99	5.07	96.8–99.0	Chr7-21109481	4.96	Chr7-21471990	5.70

**Table 4:** Genotype frequency of SDLs in F_2_ populations.

Locus/Gene	Population Size	Genotype Frequency				χ^2^ Test *	
		S6	H	S9A	NA	χ^2^	*p*
*SDL1.1*	96	21	55	13	7	2.0625	0.3566
*SDL1.2*	96	20	44	24	8	0.3636	0.8338
*SDL2*	96	24	49	23	0	0.0625	0.9692
*SDL5*	96	16	56	22	2	4.2128	0.1217
*SDL7*	96	26	45	25	0	0.3958	0.8204
*Rf-1*	96	46	43	0	7	0.1011	0.7505

S6, homozygous for Shenhui26; H, heterozygote; S9A, homozygous for *Shen9A*; *NA*, genotype data not available; *, χ^2^ test of deviation from the expected 1:2:1 ratio of S6:H:S9A genotypes for *SDL1.1*, *SDL1.2*, *SDL2*, *SDL5*, and *SDL7* loci, as well as the 1:1 ratio of S6:H genotypes for the *Rf-1* locus.

**Table 5:** The epistatic interaction (EPI) loci (*p* < 0.001) identified in the DH population.

EPI	Locus 1					Locus 2						χ^2^ Test
	Chr.	Start		End		Chr.	Start		End		χ^2^	*p*
		Marker	Position	Marker	Position		Marker	Position	Marker	Position		
*EPI-1*	1	Chr1-9618113	9618113	Chr1-10692179	10692179	5	Chr5-18996450	18996450	Chr5-19448955	19448955	24.23	1.51 × 10^−5^ *
*EPI-2*	1	Chr1-35221655	35221655	Chr1-37122278	37122278	10	Chr10-13369804	13369804	Chr10-17052933	17052933	22.25	4.06 × 10^−5^ *
*EPI-3*	1	Chr1-6789448	6789448	Chr1-7814575	7814575	12	Chr12-2938653	2938653	Chr12-4453458	4453458	36.46	3.3 × 10^−8^ *
*EPI-4*	1	Chr1-6789448	6789448	Chr1-7814575	7814575	12	Chr12-16416363	16416363	Chr12-21784888	21784888	45.61	3.37 × 10^−10^ *
*EPI-5*	1	Chr1-9341775	9341775	Chr1-10692179	10692179	12	Chr12-16416363	16416363	Chr12-21784888	21784888	30.11	7.98 × 10^−7^ *
*EPI-6*	5	Chr5-18500584	18500584	Chr5-20940019	20940019	11	Chr11-6478662	6478662	Chr11-8993081	8993081	25.79	6.91 × 10^−6^ *

* indicates a significant association between two loci (*p* < 0.001).

**Table 6:** Genotype frequency of two-locus interaction combinations.

Epistatic Interaction	SNP1	SNP2	Genotype Frequency				χ^2^ Test	
			I/II	i/ii	I/ii	i/II	χ^2^	*p*
*EPI-1*	Chr1-10115270	Chr5-19448955	55	19	97	53	54.642857	8.1828 × 10^−12^ *
*EPI-2*	Chr1-37122278	Chr10-17052933	50	26	68	80	29.571429	0.000002 *
*EPI-3*	Chr1-6960979	Chr12-4202216	97	52	67	8	73.607143	7.2044 × 10^−16^ *
*EPI-4*	Chr1-7624356	Chr12-21335477	86	58	79	1	79.607143	3.7265 × 10^−17^ *
*EPI-5*	Chr1-9618113	Chr12-21784888	82	58	76	8	60.428571	4.7608 × 10^−13^ *
*EPI-6*	Chr5-18500584	Chr11-7657093	62	89	41	32	34.392857	1.6367 × 10^−7^ *

SNP1 and SNP2 were the most significant associated SNP pair of each epistatic interaction (EPI); I and II were homozygous for Shenhui26 allele at SNP1 and SNP2 loci, respectively; i and ii were homozygous for Shen9A allele at SNP1 and SNP2 loci, respectively; * indicates a significant deviation from expected 1:1:1:1 segregation ratio (*p* < 0.01).

## Data Availability

Data are contained within the article.

## Supplementary Materials

The following supporting information can be downloaded at https://www.mdpi.com/article/10.3390/genes14112086/s1, Supplementary Table S1: Statistical summary of GBS in each sample; Supplementary Table S2: Statistical summary of homozygous ratio of GBS site in each sample; Supplementary Table S3: Genotypes of 6659 high-quality SNPs in each DH line and their parents; Supplementary Table S4: Genotypes and genetic positions of 470 SNPs in each DH line and their parents; Supplementary Table S5: LOD value of segregation distortion analysis and Chi-square test of each SNP marker in the DH population.
